# Development and content validity of an instrument for assessing the motivation for weight loss in adolescents with overweight and obesity

**DOI:** 10.1371/journal.pone.0242680

**Published:** 2020-11-25

**Authors:** David Franciole Oliveira Silva, Angélica Luiza de Sales Souza, Jéssica Bastos Pimentel, Thatyane Oliveira Souza, Eduarda Pontes dos Santos Araújo, Karine Cavalcanti Maurício Sena-Evangelista, Ricardo Fernando Arrais, Severina Carla Vieira Cunha Lima

**Affiliations:** 1 Postgraduate Program in Nutrition, Federal University of Rio Grande do Norte, Natal, Rio Grande do Norte, Brazil; 2 Postgraduate Program in Health Sciences, Federal University of Rio Grande do Norte, Natal, Rio Grande do Norte, Brazil; 3 Department of Nutrition, Federal University of Rio Grande do Norte, Natal, Rio Grande do Norte, Brazil; 4 Department of Pediatrics, Pediatric Endocrinology Unit, Federal University of Rio Grande do Norte, Natal, Rio Grande do Norte, Brazil; Pennington Biomedical Research Center, UNITED STATES

## Abstract

**Objective:**

To develop and validate the content of an instrument for assessing the motivation for weight loss in adolescents with overweight and obesity.

**Methods:**

The development and validation of the instrument were conducted in five stages, for which two systematic reviews were conducted. The first one, for the identification of questionnaires assessing the motivation for weight loss, included six studies and contributed to the selection of the domains for the instrument. The second one, conducted to identify the motivations for weight loss in adolescents with overweight and/or obesity, included six studies and contributed to the selection of 17 motivations included in the initial version of the instrument. The motivations most commonly identified were: having better health, improving my appearance, improving my self-esteem and avoiding bullying. The content validity was confirmed by a committee of 12 experts from the areas of nutrition, endocrinology, psychology, and physical education. Based on these evaluations, the content validity index was calculated. Only the items with a content validity index >0.80 for practical relevance were held in the instrument.

**Results:**

Five of the 17 motivations included in the initial version of the instrument were excluded because they had content validity index <0.80 for practical relevance. Of the 12 items held in the instrument, five were revised by experts in order to standardize wording and make the language more appropriate for adolescents. Experts categorized the items into the health, personal satisfaction, appearance and social domains.

**Conclusions:**

This is the first instrument assessing the motivation for weight loss in adolescents with overweight and obesity in Brazil. The content validity evaluation by the panel of experts provided more practical relevance, as well as contributed to a better presentation of the items. Further psychometric testing is needed to determine reliability and construct validity of the instrument.

## Background

Currently, obesity in adolescence is a serious public health problem both in Brazil and in other countries [1, 2]. According to recent worldwide estimates, over 340 million children and adolescents aged 5–19 years presented with overweight or obesity issues in 2016 [2]. In this context, the reduction and control of body weight is an important measure in order to reduce health risks in adolescence and adulthood [3]. Studies have found that many adolescents with overweight and obesity seek to lose weight on a number of grounds [4–6].

However, evidence suggests that adolescents with motivation for weight loss related to appearance or bullying avoidance are more likely to adopt unhealthy behaviors for weight loss than adolescents seeking to improve their health [7, 8]. This may lead to a weight cycling process, which is characterized by weight loss and regain in a short period and increase the risk of eating disorders [9–11]. Thus, success in weight loss and control may be related to the motivation for weight loss, i.e., the reasons that lead people to seek to lose and control weight [9, 12, 13]. In this context, identifying the motivation for weight loss can be another measure to help in the identification of the best treatment strategy for weight loss in this public.

Validated instruments for assessing motivation for weight loss addressed to various age groups have been found in the literature, however, validated instruments for adolescents with overweight and obesity are still scarce [14–19]. In studies using non-validated instruments of motivation for weight loss in adolescents with overweight and obesity, the main motivations identified were: improving health, improving appearance and avoiding bullying [12, 13, 20–23].

Thus, the present study aims to develop and validate the content of an instrument to assess the motivation for weight loss in adolescents with overweight and obesity.

## Methods

This is a methodological study to develop and validate the content of an instrument for assessing the motivation for weight loss in adolescents with overweight and/or obesity. The present research was approved by the Research Ethics Committee of the Onofre Lopes University Hospital (Brazil) (CAAE no. 56763716.7.0000.5292). Form of consent obtained: electronically via Google forms.

### Instrument development stages

The development and content validation of the instrument was conducted in five stages: 1st) definition of the construct; 2nd) properties of the construct; 3rd) identification of theoretical dimensionality; 4th) selection of items; 5th) theoretical analysis of the items—content validity. These stages are equivalent to the first five stages of the nine of the psychometric instrument development model proposed by Pasquali (1999) [24].

#### Construct development

The construct (stage 1) of the instrument developed in the present study “motivation for weight loss” was defined by us as any reasons/motives that lead someone to seek ways to lose weight. The target population of the instrument is adolescents with overweight and obesity.

The properties of the construct (stage 2), i.e., the motivations for weight loss in adolescents with overweight and obesity, were identified through systematic literature in the Scopus, PubMed, Latin American and Caribbean Health Science Literature (LILACS) and ADOLEC databases [25]. In this systematic review [25], no validated instruments for assessing motivation for weight loss in adolescents were found. Thus, the six studies included in this systematic review used questionnaires or interviews with open questions. In these studies, 17 motivations for weight loss were identified: having better health [8, 20–22], improving my appearance [8, 9, 21], improving my self-esteem [8, 9, 22], avoiding teasing/bullying [9, 15, 23], being healthier [9, 22], presenting a good physical body [9, 22], being accepted by my friends and classmates [8, 23], wearing “normal size,” “more elegant” clothes [9, 20], being more attractive/more desirable [21], doing more things to have fun [8], facing major changes in my life (going to another school or city) [8], personal motivation [8], celebrating my 15th birthday [20], to move more easily and practice physical activities [20], to feel good [22], accepting my body [22], and improving my quality of life [9].

The theoretical dimensions of the instrument (stage 3), i.e., the name given to the groups of motivations for weight loss, were identified through another systematic literature review [26]. Considering that there are no validated instruments for adolescents, this systematic review included five questionnaires assessing the motivations for weight loss of individuals with overweight and obesity in any age group. Three main domains were identified in the five questionnaires included in the review: health, appearance and social factors. Considering that some of the 17 motivations identified in stage 2 were not related to health, appearance or social domains, we also included personal satisfaction in the instrument. The conceptual framework on motivations for weight loss in adolescents with overweight and obesity is presented in S1 Fig. As this figure shows, the framework has four factors/domains related to motivations for weight loss. Health status, personal satisfaction, and body image are individual factors. The relational/social factors have a strong influence on individual factors and include relationship with family and peers and bullying.

#### Item selection

In stage 4, selection of items for inclusion in the initial version of the instrument, all 17 motivations for weight loss identified in the systematic review conducted by Silva et al. [25] were held. For item formulation, we considered aspects such as clear, objective and appropriate language to the adolescent population, presenting the necessary explanation so as not to miss the key point of the motivation, but without exaggerating the number of words to the point of hindering the understanding of the motivation.

#### Content validation

Stage 5 consisted of analyzing the content validity of the instrument. Content validity can be understood as the degree to which the items of an instrument adequately represent the construct to be measured [27]. The content validation of the instrument was made in three rounds of evaluations by a panel of experts from the areas of nutrition, physical education, psychology, and endocrinology. Polit, Beck & Owen (2007) [28] recommend that eight to 12 experts participate in the first round of evaluation. In this study, we defined that in the first round of evaluation 12 experts would be included, three of each area. According to Polit, Beck & Owen, (2007) [28], in the remaining rounds, fewer experts could be included, with a minimum of three experts per round of judging. The selection criteria for participating in the rounds with a smaller number of experts include those who respond in a shorter time, as well as those who present more suggestions.

The experts were selected using non-probabilistic intentional sampling, considering the following aspects: minimum academic qualification of specialization or residence, with least 50% of the judges (six) having doctor’s degree; and experience in clinical practice with adolescents with overweight and obesity or teaching subjects that address the theme for at least 1 year [29].

The experts were contacted by e-mail, receiving the Invitation letter to participate in the research, which included explanations about the research. After being accepted to participate in the research, they received, also by e-mail, a link to a Google form containing the Informed Consent Form (ICF), fields for personal characterization, and the checklist for evaluation of the instrument. The Google form was constructed so that experts would only have access to the instrument evaluation checklist after agreeing with the ICF.

The experts evaluated the items individually, analyzing the relevance (practical relevance) of the items in representing motivations for weight loss in the adolescent public, as well as the clarity of language. For the practical relevance of the items, analyzed in the first round of evaluation, the response options were: 1 = none, 2 = little, 3 = moderate, 4 = total. For clarity of language, analyzed in the three rounds of evaluation, the experts attributed for each item scores ranging from 1 to 4 in a Likert-type scale, being 1 = not relevant, 2 = item needs major revision to be relevant, 3 = item needs small revision to be relevant, 4 = relevant item. For items scored by experts as requiring revision, modifications could be suggested to improve the wording, making the item clearer.

The degree of agreement of the experts was calculated using the Content Validity Index (CVI) [28]. The CVI of each item, both for practical relevance and for clarity of language, was calculated by the ratio of the number of responses “3” or “4” in relation to the total number of responses to the item, in this case, 12 experts. Only the items with a CVI >0.80 for practical relevance were held in the instrument [28, 30, 31]. The assessment of language clarity used the same cut-off point of agreement among experts (CVI>0.80) and contributed to improving the presentation of the item in terms of clarity and objectivity to the target audience, adolescents. The items with CVI < 0.80 for clarity of language, were resubmitted for evaluation of the experts until they had CVI >0.80.

In addition to evaluating language clarity and practical relevance of the items, experts also categorized the items (motivations) into four domains: health, appearance, social and personal satisfaction in the first round of evaluation. When there was a tie in the number of experts who assigned an item to a domain, the highest academic qualification of the experts who chose these two domains was used as a tie-breaking rule. For example, if six experts classified one item as being part of the health domain and another six as being part of the personal satisfaction domain, the tie-break rule was the highest academic qualification.

## Results

**Fig 1:**
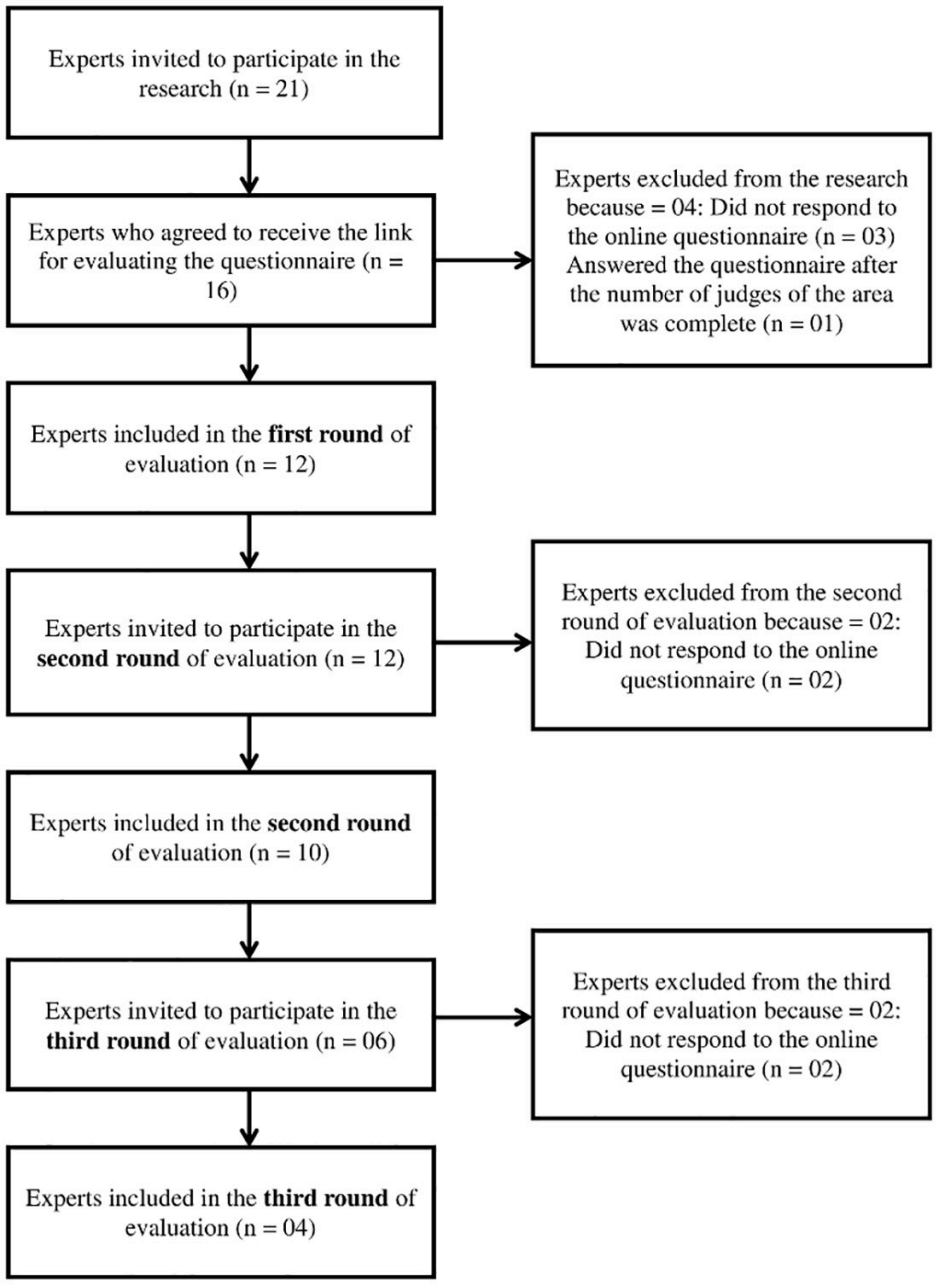
Flowchart of selection of experts to participate in content validity analysis.

Twenty-one experts were invited to participate in the research, of whom 16 accepted to receive the link to participate in the content validity analysis of the instrument (Fig 1). Of the 16 experts who received the link for evaluation of the instrument, four were excluded from the study, three for not responding to the online questionnaire about the instrument and 1 (one) for responding after the number of experts in the corresponding area was already completed. Thus, in the first round of evaluation, the responses of 12 experts were included. In the second round of evaluation, the 12 experts were again invited to respond to the questionnaire, but two did not send their answers. In the third round of evaluation, we used a sub-sample from the 12 experts included in the first evaluation round to optimize the return time of the responses, inviting experts who responded in less time and/or presented suggestions for the formulation of the items. This time, of the six experts, invited, four responded.

The characteristics of the 12 judges experts in the study are shown in Table 1. There was a higher percentage of female experts (58.3%), aged between 50 and 60 years (58.3%). Eight experts (66.6%) had a PhD degree as their highest academic degree, 75.0% had at least 15 years of professional experience, and 66.7% had worked in a public university.

**Table 1:** Characteristics of the experts included in the study (n = 12).

Features	Round 1	Round 2	Round 3
N	12.0	10.0	4.0
**Gender**			
Male	5.0 (41.7%)	4.0 (40.0%)	1.0 (25.0%)
Female	7.0 (58.3%)	6.0 (60.0%)	3.0 (75.0%)
**Age (years)**			
≥ 30 <40	2.0 (16.7%)	1.0 (10.0%)	0.0 (0.0%)
≥ 40 <50	3.0 (25.0%)	3.0 (30.0%)	2.0 (50.0%)
≥ 50 <60	7.0 (58.3%)	6.0 (60.0%)	2.0 (50.0%)
**Academic training**			
Nutritionist	3.0 (25.0%)	3.0 (30.0%)	2.0 (50.0%)
Physical educator	3.0 (25.0%)	2.0 (20.0%)	0.0 (0.0%)
Psychologist	3.0 (25.0%)	2.0 (20.0%)	2.0 (50.0%)
Endocrinologist	3.0 (25.0%)	3.0 (30.0%)	0.0 (0.0%)
**Highest academic degree**			
Specialization	2.0 (16.7%)	1.0 (10.0%)	0.0 (0.0%)
Master’s	2.0 (16.7%)	2.0 (20.0%)	1.0 (25.0%)
PhD degree	8.0 (66.6%)	7.0 (70.0%)	3.0 (75.0%)
**Professional working time (years)**			
<10	2.0 (16.7%)	1.0 (10.0%)	0.0 (0.0%)
≥10 <15	1.0 (8.3%)	1.0 (10.0%)	0.0 (0.0%)
≥15 <20	1.0 (8.3%)	1.0 (10.0%)	0.0 (0.0%)
≥20	8.0 (66.7%)	7.0 (70.0%)	4.0 (100.0%)
**Workplace**			
Public university	8.0 (66.7%)	7.0 (70.0%)	2.0 (50.0%)
Private clinic	3.0 (25.0%)	2.0 (20.0%)	1.0 (25.0%)
Public hospital	1.0 (8.3%)	1.0 (10.0%)	1.0 (25.0%)

**Source:** The authors (2020)

**Fig 2:**
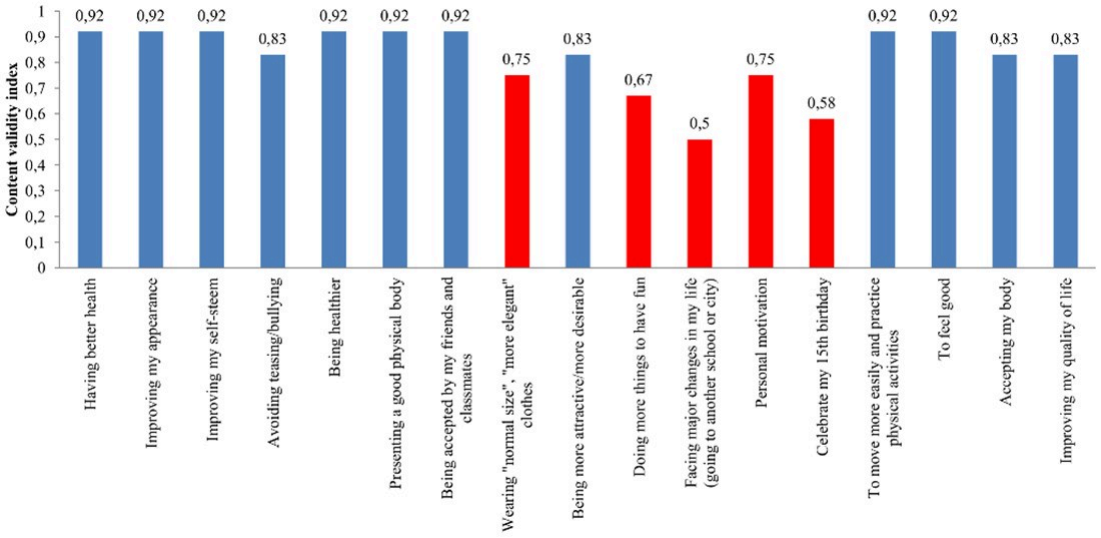
CVI for relevance (practical relevance) of the instrument items, in the first round of evaluation. Blue bars: items with CVI ≥ 0.80—held in the instrument; Red bars: items with CVI <0.80—excluded from the instrument.

Fig 2 presents the CVI for practical relevance of the 17 items analyzed by the experts in the first round of evaluation. Eight items obtained 92% agreement among the experts (CVI = 0.92) for practical relevance, with no agreement of 100% for any item. Five items evaluated for practical relevance with CVI <0.80 were excluded: wearing “normal size,” “most elegant” clothes; doing more things to have fun; facing major changes in my life (going to another school or city); personal motivation; and, celebrating my 15th birthday.

**Fig 3:**
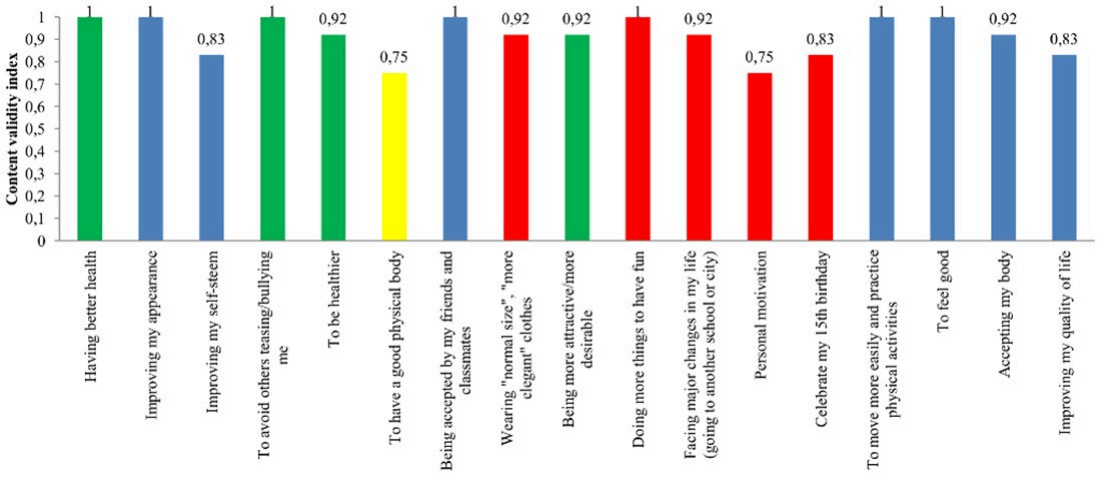
CVI for clarity of language of the instrument items, in the first round of evaluation. Blue bars: items with CVI ≥ 0.80 for clarity of language—held in the instrument without undergoing revision; red bars: items with CVI <0.80 for practical relevance—excluded from the instrument regardless of CVI for language clarity; yellow bar: item with CVI <0.80 for clarity of language—held in the instrument after revision of the wording; green bars: items with CVI ≥ 0.80 for clarity of language that even so had their wording revised according to the judges’ suggestions.

The CVI for language clarity of the 17 items of the initial version of the questionnaire is presented in Fig 3. Seven items obtained 100% agreement among the experts for language clarity. Only the item “presenting a good physical body” had a CVI <0.80 and was revised, according to the experts’ suggestions, to “having a good physical body”.

In the second round of evaluation, in which the instrument was evaluated by ten experts, the item “having a good physical body” had CVI = 0.70 and was sent to a new appreciation by the specialists. In the third round of evaluation, the item was changed to “have a healthy body,” a suggestion from one of the experts, which was accepted by all four experts who answered the online evaluation questionnaire in this round.

The CVI for clarity of language of the items: “having better health”, “avoiding others teasing/bullying me”, “being healthier” and “being more attractive, more desirable” was ≥0.80. However, even with the score higher than the established cutoff point, these items were revised considering the suggestions of experts, to make the language more appropriate to the public of the instrument and to standardize the style of writing with the other items. These items are highlighted in green in Fig 3. The wording of the following items was modified: from “having better health” to “improving my health”; from “avoiding teasing/bullying” to “avoiding others teasing/bullying me”; from “being healthier” to “to make me healthier”; and, from “being more attractive/more desirable” to “me being more attractive/more desirable.” After the evaluation of 10 experts in the second round of evaluation, all these items had CVI ≥0.80 for language clarity, and then the modifications were maintained. At the end of content validity, the instrument has a total of 12 items (S1 Appendix).

**Fig 4:**
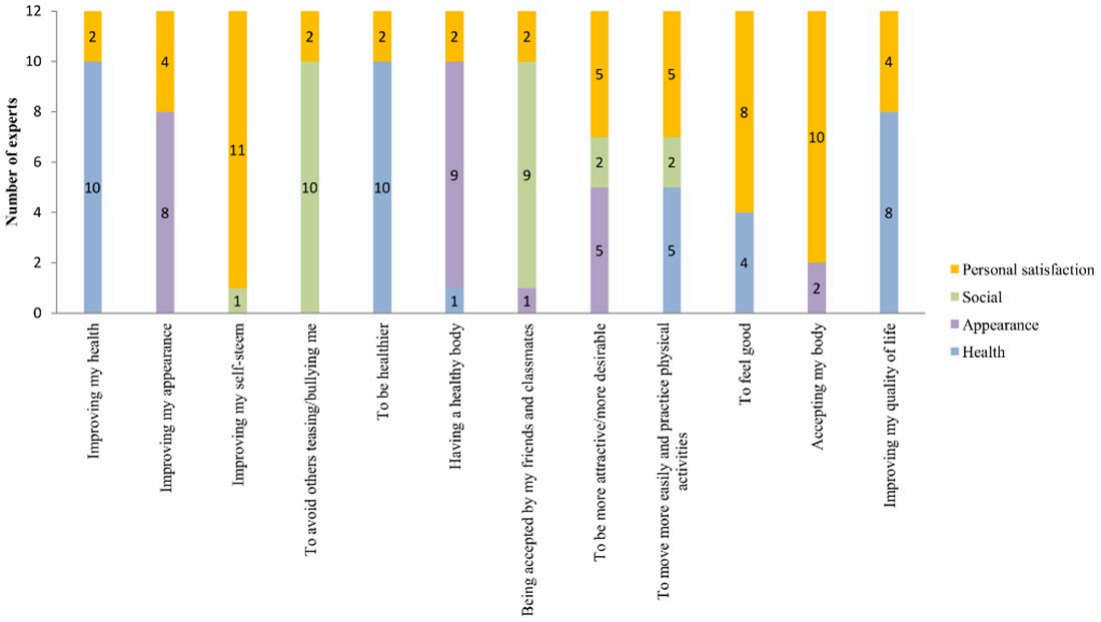
Categorization of the items of the instrument into domains by the judges.

The categorization of the 12 items into the four domains by the experts is presented in Fig 4. The item “to be more attractive/more desirable” was classified by five experts as belonging to the appearance domain and five other experts considered it as belonging to the personal satisfaction domain. Of the five experts that classified the item as belonging to the personal satisfaction domain, four had at least 15 years of professional experience and one had between 10 and <15 years of professional experience, while of the five experts that classified the item as belonging to the domain appearance, three had at least 15 years of professional experience and two had < 10 years of professional experience. For that reason, the item was included in the personal satisfaction domain. Likewise, there was a tie in the number of experts who classified the item “to move more easily and practice physical activities” as belonging to the health and personal satisfaction domains. Considering the professional experience time, the item was included in the personal satisfaction domain, since five experts with at least 15 years of professional experience chose this domain, while three experts with at least 15 years of professional experience chose the health domain.

In the end, three items were included in the health domain (improving my health, to be healthier, and improve my quality of life), five were included in the personal satisfaction domain (improving my self-esteem, to be more attractive/more desirable, to move more easily and practice physical activities, to feel good and accept my body), two were included in the appearance domain (improving my appearance and having a healthy body), and two were included in the social domain (to avoid others teasing/bullying me, and being accepted by my friends and classmates).

## Discussion

In the present study, an instrument for assessing the motivation for weight loss in adolescents with overweight and obesity was developed and validated. The 12-item instrument, to the best of our knowledge, is the first with items and text aimed at teenagers. In order to evaluate content validity, experts from four different professional areas, namely, nutrition, physical education, psychology, and endocrinology, which are the health professionals most involved in the treatment for weight loss in adolescents with overweight and obesity, were included.

The number of experts in each round of evaluation was different. It is noteworthy that in the second round we tried to include the same number of experts of the first round given that five items were revised and sent for analysis of the experts. The inclusion of a smaller number of experts in the third round is in line with the recommendations of Polit, Beck & Owen (2007) [28], which suggest that at least three specialists participate in each stage, from the second round. Thus, the reduced number of experts in the third round does not correspond to a sample loss, but rather a reduction to optimize the response time, considering that in the third round only one item was revised.

The evaluation of the relevance (practical relevance) of each item/ motivation of the instrument was a crucial stage for the development of this instrument since in this stage we selected the 12 items/motivations that were considered of great practical relevance for the adolescent public by the panel of specialists. It should be noted that the 17 motivations initially evaluated by the specialists were identified in studies conducted on adolescents with overweight and obesity, although using instruments were not validated [8, 9, 20–23]. Thus, the 12 items held in the instrument represented motivations identified in the literature and observed in the practice of specialists. This reduction in the number of items may also favor the moment of application of the instrument, considering that the target audience of the instrument are adolescents.

The inclusion of the item “improving my quality of life” is particularly relevant, as studies have found that adolescents with overweight and obesity issues report a lower quality of life when compared to adolescents with eutrophic BMI [32, 33]. With this, the professional approach should enhance the follow-up of healthy weight control behaviors, for the promotion of quality of life and health [34].

Adolescents with overweight and obesity issues may present with higher body dissatisfaction [35, 36]. In this sense, the inclusion of the item “improve my appearance” can contribute to early multi-professional intervention that considers adolescents’ desire to improve their appearance, but also explains to them diversity in body shapes, especially during adolescence, a period in which there are significant body changes [37]. Furthermore, when looking for rapid weight loss, observed when teenagers want to improve their appearance, it is important to monitor unhealthy weight control behaviors that can lead to unsatisfactory results and compromise the health status of the teenager [38, 39].

Provocation and bullying, including those related to body weight, are frequent in adolescents with overweight and obesity issues [8]. Thus, the identification of a motivation for weight loss to avoid provocation and bullying can favor the integrality of health care, when considering psychological and social factors in the treatment for weight loss in the adolescent population [3]. Furthermore, the identification of this motivation can contribute to the establishment of inter-sectoral measures, including those related to schools, for the promotion of healthy relationships based on respect for human dignity, cultural diversity, and the plurality of ideals and thoughts, and not related to body weight or shape of adolescents [40, 41].

Among the items/motivations excluded from the instrument, only “wearing normal size, more elegant clothes” was identified in two studies [9, 20] in the literature review conducted by Silva et al. [25] to identify the motivations for weight loss in adolescents with overweight and obesity. The motivations excluded: doing more things to have fun [9]; facing major changes in my life (going to another school or city) [9]; personal motivation [9]; and, celebrating my 15^th^ birthday [20], were observed, each one, in only one study in the systematic review [25].

Analyzing the clarity of the language of the items of an evaluation instrument is very important for minimizing possible directives and confusion in the process of instrument application [42]. The experts’ suggestions for revising the items were analyzed by considering the frequency of suggestions as well as to make the items standardized in terms of wording. Thus, although only one item had an unsatisfactory evaluation (CVI <0.80) for language clarity: “presenting a good physical body,” four other items were also revised with the aim of standardizing the wording of the items and making the language more appropriate for the adolescent public. These modifications were submitted for analysis and were accepted by the panel of experts.

The categorization of the instrument items into domains was made by the panel of specialists which had different academic backgrounds. The nutritionists were the ones who most indicated items as belonging to the personal satisfaction domain (five), while the psychologists and endocrinologists classified two items as belonging to this domain. The psychologists indicated five items as belonging to the health domain, while the nutritionists indicated only three items as belonging to this domain. The nutritionists and physical educators indicated two items as belonging to the appearance domain, while psychologists and endocrinologists indicated three. The social domain was the only domain which obtained agreement among the professionals from the four areas of academic training, indicating the inclusion of two items in this domain.

Considering that the categorization of the items into domains was based on the indications of professionals from four different academic backgrounds (nutritionists, psychologists, physical educators, and endocrinologists), the categorization included different knowledge and professional contexts. Thus, after the categorization of the items, the instrument was well distributed as to the number of items in each domain, especially the motivations of the health and personal satisfaction domains, with three and five motivations each, respectively.

This instrument for evaluating the motivation for weight loss in adolescents with overweight and obesity differs from that used for the general public by the inclusion of the item “avoiding others teasing/bullying me”, not found in other instruments [15–19], as well as by the non-inclusion of the motivation “to improve my work performance”, found in instruments for adults [16, 17], with little applicability to the adolescent public. The relevant contribution of the specialists for adapting the language used in the wording of the items for the adolescent public is noteworthy, for example, the item “accepted by my friends and schoolmates”, is usually found in the instruments for the general public as: “to be more successful with others” [16, 17], “to be accepted by society” [16, 17].

One of the limitations of this study is that the instrument was validated only as to its content. However, the instrument developed in this study will be evaluated as to construct validity and reliability in the future. In addition, there are several published studies that only present content validation, considering the importance of describing in detail the initial stages of development of the instruments [43–45]. The positive aspects of this study include: 1) novelty of work, since, to the best our knowledge, there is no current validated instrument for the evaluation of motivation for weight loss in adolescents with overweight and obesity; and 2) the inclusion of specialists with relevant professional and/or academic experience to evaluate the content validity of the instrument.

## Conclusions

The instrument that we developed and validated is the first one to evaluate the motivation for weight loss in adolescents with overweight and obesity. The evaluation of the items by the panel of experts allowed the selection of the most practical items for adolescents. Furthermore, the revisions made in the wording of the items provided a better standardization in the presentation of the items and a clearer language adapted to the age range of the target audience.

## Supporting information

S1 Fig Conceptual framework on motivations for weight loss in adolescents with overweight and obesity. (TIF)

S1 Appendix Instrument for assessing the motivation for weight loss in adolescents with overweight and obesity. (DOCX)
