# Supplementary figures and images for: Development and content validity of an instrument for assessing the motivation for weight loss in adolescents with overweight and obesity
Source: PLoS One. 2020 Nov 25;15(11):e0242680. doi: 10.1371/journal.pone.0242680 (PMC7688166; doi:10.1371/journal.pone.0242680)

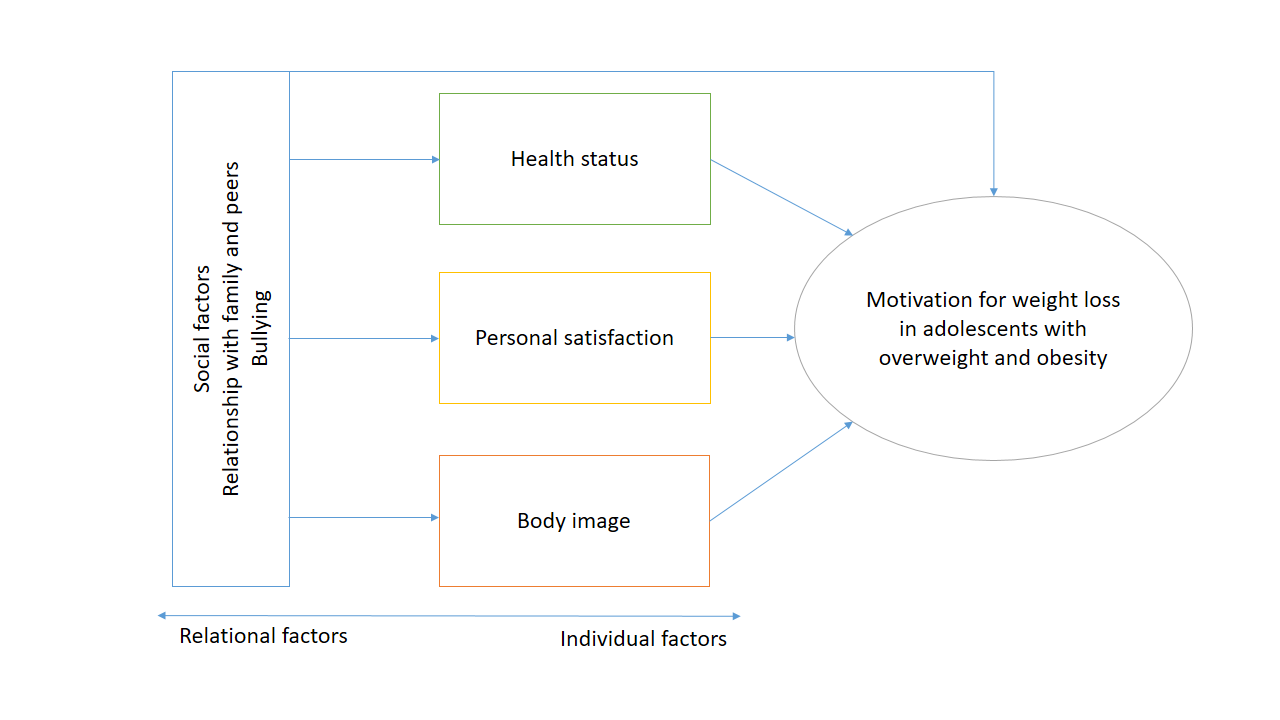

Supplement: S1 Fig — (TIF) [file pone.0242680.s001.tif]
