# Supplementary material for: Development and content validity of an instrument for assessing the motivation for weight loss in adolescents with overweight and obesity
Source: PLoS One. 2020 Nov 25;15(11):e0242680. doi: 10.1371/journal.pone.0242680 (PMC7688166; doi:10.1371/journal.pone.0242680)
Supplement: S1 Appendix — (DOCX) [file pone.0242680.s002.docx]

**S1 Appendix. Instrument for assessing the motivation for weight loss in adolescents with overweight and obesity.**

What is the level of influence of the following items on your motivation for weight loss?

| Motivation | 0 - No influence | 1 - Little influence | 2 - Moderate influence | 3 - Lots of influence | 4 - Total influence |
| --- | --- | --- | --- | --- | --- |
| Improving my health |  |  |  |  |  |
| Improving my appearance |  |  |  |  |  |
| Improving my self-esteem |  |  |  |  |  |
| To avoid others teasing/bullying me |  |  |  |  |  |
| To be healthier |  |  |  |  |  |
| Having a healthy body |  |  |  |  |  |
| Being accepted by my friends and classmates |  |  |  |  |  |
| To be more attractive/more desirable |  |  |  |  |  |
| To move more easily and practice physical activities |  |  |  |  |  |
| To feel good |  |  |  |  |  |
| Accept my body |  |  |  |  |  |
| Improve my quality of life |  |  |  |  |  |

**Apêndice 1. Intrumento de avaliação da motivação para a perda de peso em adolescentes com sobrepeso e obesidade**

**Qual o nível de influência dos itens que se seguem em sua motivação para perda de peso?**

| Motivação | 0 – Nenhuma influência | 1 – Pouca influência | 2 – Moderada influência | 3 – Muita influência | 4 – Total influência |
| --- | --- | --- | --- | --- | --- |
| Melhorar a minha saúde |  |  |  |  |  |
| Melhorar minha aparência |  |  |  |  |  |
| Melhorar minha autoestima |  |  |  |  |  |
| Para evitar provocações/bullying comigo |  |  |  |  |  |
| Para eu ser mais saudável |  |  |  |  |  |
| Ter corpo saudável |  |  |  |  |  |
| Ser aceito(a) pelos meus amigos e colegas de escola |  |  |  |  |  |
| Para eu ser mais atraente/mais desejado(a) |  |  |  |  |  |
| Para me movimentar mais facilmente e praticar atividades físicas |  |  |  |  |  |
| Para sentir-me bem |  |  |  |  |  |
| Aceitar meu próprio corpo |  |  |  |  |  |
| Melhorar minha qualidade de vida |  |  |  |  |  |
